# The cortical canvas

**DOI:** 10.1073/pnas.2610120123

**Published:** 2026-05-04

**Authors:** Bevil R. Conway, Spencer R. Loggia

**Affiliations:** ^a^Laboratory of Sensorimotor Research, National Eye Institute and National Institutes of Mental Health, Bethesda, MD 20892; ^b^Neuroscience Graduate Program, Brown University, Providence, RI 02912

If you peel off the gray matter of the cerebral cortex, like the rind from an orange, you will recover a sheet of tissue. This sheet contains the neuron cell bodies responsible for sensory perception, long-term memory, problem solving, and decision-making. Understanding how it works is daunting because science typically assumes that we begin with a question, a hypothesis, which implies we can already imagine how the system might work. But our imaginations may not suffice for a theory of something as complex as the organ responsible for perception and thought. Hypothesis-free, data-driven approaches are less constrained but promise to expand our imagination. In this issue of PNAS, Pennock et al. adopt a data-driven approach, treating the cortex like a sheet of photographic film to be developed in some way, to see what you see. In this case, the authors “develop” the cortical canvas by painting each location with the average image (from many thousands) that activated it (1). What makes the cortical paintings compelling is that they point, surprisingly, to color as a tag for functional distinctions in high-level cortical areas, opening many questions.

**Fig. 1.**
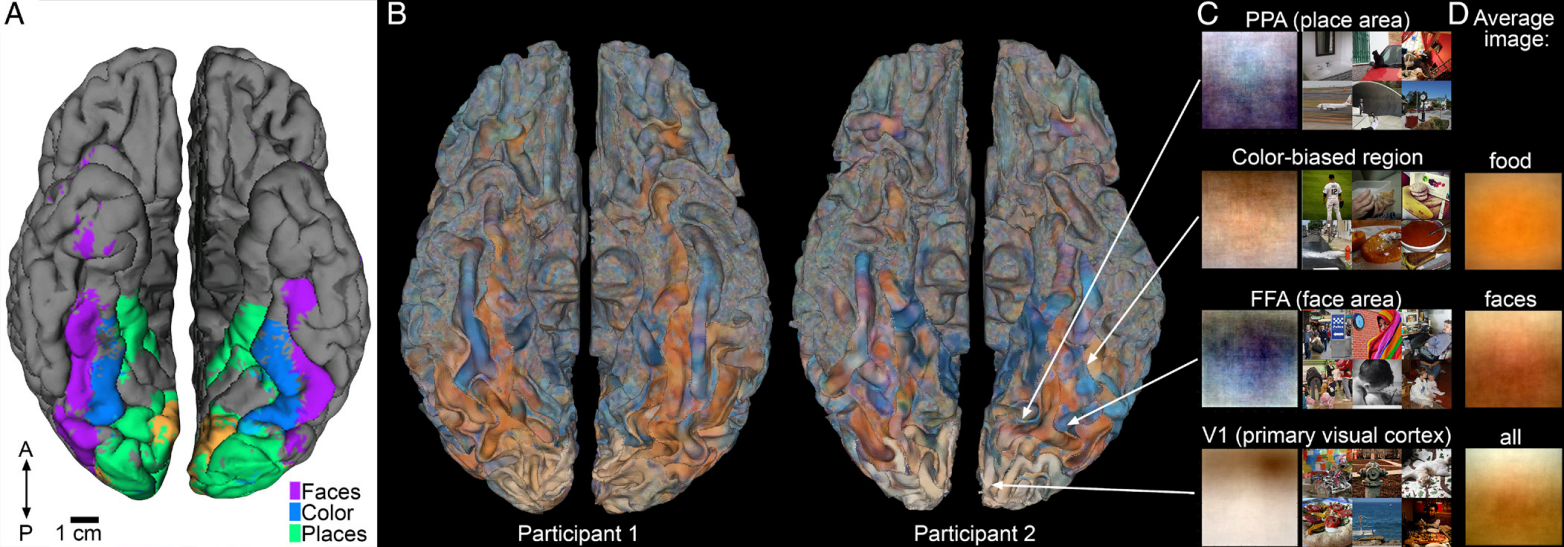
The functional organization of the ventral visual pathway recovered with conventional fMRI experiments and with a new data-driven approach—Voxel Preferred Images (VPIs). (*A*) cortical surface showing the ventral view of the human brain, aggregated data from 18 participants contrasting responses to color and grayscale images of faces, places, and objects, A is anterior, P is posterior (5). (*B*) VPI surfaces for two participants (1). (*C*) VPIs for four voxels and the six images eliciting the strongest response from each (PPA, parahippocampal place area; FFA, fusiform face area). (*D*) The average image of the Natural Scenes Dataset for food, faces, and all images. Panels *B* and *C* provided by Ian Pennock and Jenny Bosten.

Early anatomical studies pioneered data-driven methods, developing the cortex using a Nissl stain that revealed subtle variations in cell size and density across the cortex (2). These observations suggested the cortical sheet is partitioned into regions, each serving a specific function. This idea has been extensively probed, particularly with fMRI experiments that often test specific hypotheses by contrasting responses to two conditions—for instance, faces versus places. Such experiments led to the identification of the fusiform face area (3), the para-hippocampal place area (4), and color-biased regions sandwiched between them (5). Together these studies imply that high-level visual cognition is carried out on the ventral surface of the cerebral cortex in parallel channels (6) (Fig. 1*A*).

But perhaps the conventional experimental design, where responses to one condition are contrasted with responses to another, preemptively commits us to one way of thinking about how the cortex works. An alternative approach is data-driven discovery, and one rich vein of data is the Natural Scenes Dataset (NSD), a vast repository of whole-brain, high-resolution fMRI measurements from eight participants who each viewed 9,000 to 10,000 color natural scene images over dozens of weekly scan sessions (7). Work with the NDS has afforded many new observations, including that the color-biased regions are sensitive to images of food (8, 9), perhaps because food is colorful.

Pennock et al. pick up work with the NDS, painting the cortical sheet in three steps. First, they determined the extent to which each image activated each voxel in each participant, measured as the beta coefficient for each image in a general linear model of the fMRI response. Next, they generated the average preferred image for each voxel, weighting each image’s contribution by its beta coefficient and scaling the resulting RGB values to fill the gamut. Finally, they painted each voxel of the cortical sheet with its average Voxel Preferred Image (VPI).

The most salient feature of these maps is reproducible patches of color (Fig. 1*B*). Some of the patterns are consistent across people, others are individual specific; their reproducibility shows they are meaningful. In the VPI-painted cortex, the occipital pole (the posterior end), which houses primary visual cortex (V1), is distinctively pale across participants. Zooming in shows that the individual. VPIs contain a noticeable dark blob (Fig. 1*C*). From these results, we learn that the average image for V1 is dominated by a dark thing on a light background, which provides an intriguing hypothesis for the OFF-dominated scaffolding that organizes V1 (10, 11). The average VPI is not obvious from the images that most strongly activate V1 (Fig. 1*C*). Is it apparent in an average of *all* the images in the Natural Scenes Dataset? We looked, and it is not: the average image is shades of brown with a fuzzy horizon forming a hazy landscape (Fig. 1*D*). So, the VPI images uncover an unexpected image-selection operation of V1.

In PNAS, Pennock et al. adopt a data-driven an approach, treating the cortex like a sheet of photographic film to be developed in some way, to see what you see.

Emanating from V1, the VPI-painted cortex shows bands dominated by blue and orange (Fig. 1*B*), the main colors of scenes. The bands roughly match the functional partitions discovered with more conventional tests (compare with Fig. 1*A*), but only roughly, and the colors of the VPIs are surprising. VPIs of voxels in face areas are often blue—this is surprising because human faces of all races are relatively warm-toned compared to their backgrounds. Are faces in the NDS on average blue? Again, we looked, and they are not—they have the expected warm coloring (we selected NSD images for “people” + “cellphone” to identify images of faces). The blue color of many face-area VPIs raises an unexpected question: are these areas sensitive to people-in-context? People like blue. This is evident in the most-preferred images for the FFA voxel in Fig. 1, where four of the images show people wearing blue.

The VPIs of the PPA, meanwhile, tend to be bottom-heavy, consistent with natural scenes where ground is dark and sky is light, but they are also often blue-dominated, which is another surprise given the average of all images (“places” in the NDS) is orange. The VPIs of voxels in color-biased regions tend to be warm-colored, top-heavy fields, consistent with the color statistics of objects (12) and the typical location of objects in the upper visual field during natural behavior. But again, there are noticeable differences between the VPIs and expectations from average images in the dataset: the average image of food is not top-heavy and is more saturated orange (Fig. 1*D*). Many of the top images for color-biased voxels contain food, but not all of them, suggesting the region’s job may not be to identify food, but rather to use color to tag behavioral relevance more generally.

What explains the colors of the VPIs? Why does any VPI ever appear white or blue if the average image of any putatively prioritized category of objects is not blue or white? As shown by examining the VPIs of face, place, and color-biased regions, the VPI approach flips the traditional script: rather than using brain measurements to teach us how the cortex implements an operation, it raises questions about the operations themselves.

But the method also has the potential to shed light on old puzzles of brain organization. In macaque monkeys, area V4 consists of dorsal and ventral parcels representing the lower and upper visual fields. The corresponding cortical region in humans, dubbed “hV4” to mark the species difference, appears to have only a ventral parcel that may represent only the upper visual field (reviewed in ref. 13). Pennock et al. show that the patchy VPI color structure seen in hV4, below ventral V3, reappears above dorsal V3, suggesting that hV4 and macaque V4 are more similar than different. Importantly, the authors show that representational dissimilarity matrices of voxel clusters with similar VPI colors are significantly more similar for same-color clusters than for different-color clusters, even when cortical distance is matched, indicating that the color structure of the VPI maps reflects real functional divisions.

The VPI method has the potential to make new discoveries about brain organization, too. One participant shows a pink patch in the medial superior frontal cortex overlying parcels SFL, 8BM, and SCEF in the Human Connectome Project. Little is known about these parcels, although they are thought to straddle language, motor, and salience systems. The corresponding VPIs in this person are pink, dominated by babies and children, and distinguished from nearby blue patches, which suggests the region connects salience, affect, and motor planning around a category significant for the participant. This result is striking not because “pink” is the encoded feature, but because it tags the relevant images. That not all participants showed the same VPIs in this region hints at its plasticity. It will be fun to explore VPIs in other regions, such as those implicated in theory-of-mind or aesthetic judgments.

The fact that color provides a meaningful way to map the visual cortex might be surprising—after all, much of what vision does (face recognition, object discrimination) is available in grayscale. But before the invention of photography, color was recognized as central to vision, and there has been a resurgence of interest in color given its utility in machine vision (14), the acute sensitivity of the primate visual system to chromatic changes (15), the large expanse of cortical real estate engaged by color (6), and evidence from brain-damaged patients showing that “color vision” comprises many behaviors (16), including a profound role in affect and reward. Indeed, an addiction to smart phones can be combatted by making the display grayscale (17). This resurgence of interest is evident in neuroimaging experiments that exploit color to discover functional signatures of visual areas (18, 19).

The VPI method uses color as a tag, but does it tell us anything about the brain’s processing of color? Perhaps. The ability to judge the average value of stimuli composed of distributed features is an important cognitive function, yet little is known about how the brain computes such averages for color (20)—a capacity that seems important for color constancy. The VPI method could provide insight into how the brain computes ensemble color statistics (the white of V1 VPIs may be especially instructive). But what exactly is a voxel “preferring” in a VPI? Is it hue, luminance contrast, spatial structure, or meaning linked to color? The answer is likely all of the above. This ambiguity is not a weakness of the method; it is the point of departure. The VPI method is a hypothesis-generating machine, offering compact, intuitive descriptions of what drives the cortex, and it gives us a roadmap for experiments we might not otherwise have imagined.
